# Correction: Nickel-catalyzed γ-alkylation of cyclopropyl ketones with unactivated primary alkyl chlorides: balancing reactivity and selectivity *via* halide exchange

**DOI:** 10.1039/d4ra90052a

**Published:** 2024-05-07

**Authors:** Zhen-Ying Wang, Shi-Zheng Liu, Cong Guo, Yi-Zheng Cheng, Qiang Li, Jianmin Dou, Dacheng Li

**Affiliations:** a Shandong Provincial Key Laboratory of Chemical Energy Storage and Novel Cell Technology, School of Chemistry and Chemical Engineering, Liaocheng University Liaocheng 252000 P. R. China liqiang9@lcu.edu.cn jmdou@lcu.edu.cn lidacheng62@163.com

## Abstract

Correction for ‘Nickel-catalyzed γ-alkylation of cyclopropyl ketones with unactivated primary alkyl chlorides: balancing reactivity and selectivity *via* halide exchange’ by Zhen-Ying Wang *et al.*, *RSC Adv.*, 2024, **14**, 12883–12887, https://doi.org/10.1039/D4RA02616K.

The authors regret that the name of one of the authors (Zhen-Ying Wang) was shown incorrectly in the original article. The corrected author list is as shown here.

The Royal Society of Chemistry apologises for these errors and any consequent inconvenience to authors and readers.

## Supplementary Material

